# Ten ensembles with hourly high spatial resolution datasets for severe precipitation events in Egypt

**DOI:** 10.1016/j.dib.2020.105987

**Published:** 2020-07-05

**Authors:** Muhammed Eltahan, Sabah Alahmadi, Karim Moharm, Mohammed Magooda

**Affiliations:** a Aeronautical and Aerospace Engineering Department, Cairo University, Cairo 12613, Egypt; b Now at Institute of Bio- and Geosciences (IBG-3: Agrosphere), Forschungszentrum Jülich, Jülich 52428, Germany; c Now at Centre for High-Performance Scientific Computing in Terrestrial Systems, Geoverbund ABC/J, Jülich 52428, Germany; d National Satellite Technology Center, Space and Aeronautics Research Institute, King Abdulaziz City for Science and Technology, Riyadh 11442, Saudi Arabia; e Electrical Engineering Department, Alexandria University, Alexandria 21544, Egypt

**Keywords:** High resolution meteorological dataset, Egypt, Temperature, Wind speed, Precipitation

## Abstract

In this article, we created a new dataset comprising 10 ensembles for severe rainfall simulations at a high spatial resolution (10 km grid spacing, 120 × 120 grid points) in Egypt using the Weather Research and Forecast model Version 3.8 (WRF 3.8). The vertical grid had over 41 levels, extending from the surface to 10 hPa. The defined domain, a Lambert conformal conic projection, started from 24°E to 36°E. The ensembles were generated using 10 different microphysics schemes within the WRF 3.8. The severe rainfall event occurred between October 26 and 29, 2016. Final analysis data from National Center for Environmental Predictions were used for the initial and boundary conditions every 6 h at a spatial resolution of 1° × 1°. The geographical static input data, such as land use, albedo, and terrain height, were interpolated and prepared using a geogrid program in the WRF preprocessing system. This dataset is the first of its kind. It is addressing a need for this type of high resolution data over Egypt using physically- based numerical weather prediction models.

Specifications table

**Table:** 

Subject	Atmospheric science
Specific subject area	Numerical weather modelling
Type of data	An hourly, three-dimensional, gridded dataset saved as a network Common Data Form (netCDF) format. It features information for 10 ensembles (for a rainfall event taking place from October 26 to 29, 2016) at a high spatial resolution (10 km).
How data were acquired	This dataset was generated using the Weather Research and Forecast model Version 3.8 (WRF 3.8). Source code of WRF 3.8 model can be obtained through gitlab repository (https://github.com/wrf-model/WRF). The selected configuration for the physical parameterizations is highlighted in Description of Data Collection. Further information about the model setup and configuration is provided in [9].
Data format	Raw, filtered
Parameters for data collection	The dataset consists of 14 meteorological parameters:
	RAINNC: accumulated total grid scale precipitation (mm)
	RAINSH: accumulated shallow cumulus precipitation (mm)
	RAINC: accumulated total cumulus precipitation (mm)
	U: x-wind component (m/s)
	V: y-wind component (m/s)
	W: z-wind component (m/s)
	P: perturbation pressure (Pa)
	T2: temp at 2 m (K)
	U10: U at 10 m (m/s)
	V10: V at 10 m (m/s)
	SST: sea surface temperature (K)
	QRAIN: rainwater mixing ratio (kg:kg)
	QCLOUD: cloud water mixing ratio (kg:kg)
	QVAPOR: Water vapor mixing ratio (kg:kg)
Description of data collection	The high spatial resolution dataset was generated using the WRF 3.8. The vertical grid had over 41 levels, extending from the surface to 10 hPa. The defined domain (a Lambert conformal conic projection) started from 24°E to 36°E. The 10 ensembles were generated using 10 different microphysics schemes within the WRF model: Kessler [1], Purdue–Lin [2], WRF Single-Moment-Microphysics 3-class [3], WRF Single-Moment-Microphysics 5-class, Eta Ferrier, WRF Single-Moment-Microphysics 6-class [4], Goddard [5], Thompson [6], Milbrandt–Yau Double Moment [7], and Morrison Double Moment [8]. It used RRTMG longwave and shortwave models and the Unified Noah Land Surface model. The Yonsei University planetary boundary. layer scheme and the MM5-similarity scheme were also used, in addition to the Grell 3D Ensemble (a cumulus scheme). The severe rainfall event occurred between October 26 and 29, 2016. The total precipitation from 10 ensemble are compared against precipitation from Tropical Rainfall Measuring Mission (TRMM) as shown in [9].
Data source location	Aerospace Engineering Department, Cairo University, Cairo, Egypt
Data accessibility	The data can be accessed through Mendeley at https://data.mendeley.com/datasets/js5984ckhh/1 or using the DOI, 10.17632/js5984ckhh.1.
Related research article	M. Eltahan, and M. Magooda, 2018: Sensitivity of {WRF} microphysics schemes: case study of simulating a severe rainfall over Egypt. J. Phys.: Conf. Ser. 1039 012024. https://doi.org/10.1088/1742–6596/1039/1/012024.

## Value of the data

• Egypt lacks high-resolution meteorological datasets especially the precipitation. This dataset contains 10 ensembles generated using different numerical simulations from the WRF model for a heavy rainfall event that occurred in Egypt on October 26, 2016, at hourly intervals. This dataset is considered to be unique and critical.
• This high-resolution (10 km) dataset was generated using the WRF 3.8, which consistently represents the physical processes in the atmosphere and the interaction with land surface. This dataset features three-dimensional information, which is unlike the few existing and available datasets for specific locations.
• These 10 ensembles can be used to differentiate artificial intelligence algorithms in the classification and identification of precipitation and temperature patterns for heavy rainfall events.
• This dataset can be used to determine the correlation among different meteorological datasets. Other statistical models can also be used to analyze other impacts.
• This dataset is especially useful for flagship pilot studies within the Coordinated Regional Downscaling Experiment (CORDEX) framework which is part of World Climate Research Programme (WCRP). This flagship pilot study is interested in simulating short-term heavy rainfall events using regional climate models in “weather-like” mode in different domains [10].
• This dataset can be processed and visualized easily using both Climate Data Operator (CDO) and Ncview software respectively.

## 1 Data description

The dataset was divided into 10 directories, each representing an ensemble generated using a different microphysics scheme. The directory naming convention is WRF_microphysics_scheme_xyz, where “xyz” represents the used scheme. For example, WRF_microphysics scheme_ Kessler represents an ensemble whose WRF simulation utilized the Kessler microphysics scheme.

Each directory features four NetCDF files. Each file contains data for a single day (24 h), consisting of 14 variables (see Specification Table) measured in three dimensions on an hourly basis. The file naming convention is EGYPT_WRF_3_8_xyz_dd_mm_yyyy.nc, where “xyz” represents the scheme used, “dd” is the date, “mm” is the month, and “yyyy” is the year. For example, EGYPT_WRF_3_8_ Kessler_25_10_2016.nc would refer an ensemble that utilized the Kessler microphysics scheme on October 25, 2016.

## 2 Experimental design, materials, and methods

### 2.1 Model setup

**Fig. 1:**
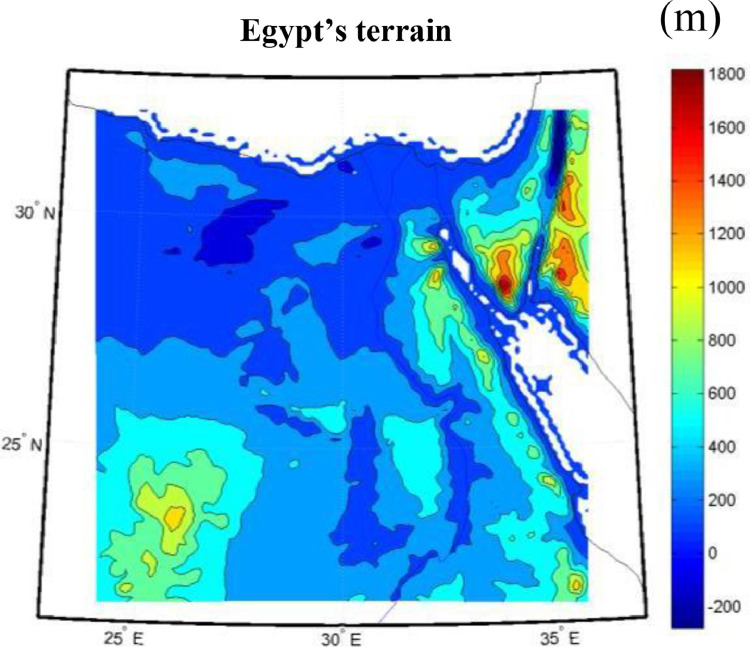
Egypt's terrain generated using the WRF.

WRF 3.8 [11] was used to simulate meteorological events over a model domain with the topography shown in Fig. 1. The model domain is a Lambert conformal conic projection that extends from 24°E to 36°E (120 grid points) and 21.5°N to 32.5°N (120 grid points), covering 100 km^2^ (10 km × 10 km). The vertical grid has over 41 levels, extending from the surface up to 10 hPa. The static geographical fields, such as terrain height, soil properties, vegetation fraction, land use, and albedo, were interpolated and preprocessed using the geogrid program in the WRF preprocessing system (WPS). Final analysis data from National Center for Environmental Predictions were used for the initial and boundary conditions every 6 h at a spatial resolution of 1° × 1° The boundary and initial conditions were interpolated based on the model domain using the ungrib and metgrid programs in the WPS.

### 2.2 Physical parameterizations

The 10 ensembles were generated using 10 different microphysics schemes within WRF 3.8: Kessler, Purdue–Lin, WRF Single-Moment-Microphysics 3-class, WRF Single-Moment-Microphysics 5-class, Eta Ferrier, WRF Single-Moment-Microphysics 6-class, Goddard, Thompson, Milbrandt–Yau Double Moment, and Morrison Double Moment. It used RRTMG longwave and shortwave models and the Unified Noah Land Surface model. The Yonsei University planetary boundary scheme and the MM5- similarity scheme were also used, in addition to the Grell 3D Ensemble (a cumulus scheme). The severe rainfall event occurred between October 26 and 29, 2016. The precipitation data of the 10 ensembles were compared to the Tropical Rainfall Measuring Mission's satellite data as presented in [9]. The source code for the WRF is available for community use community on GitHub (https://github.com/wrf-model/WRF).

## Declaration of Competing Interest

The authors have no conflicts of interest to report.

## Appendix Supplementary materials

Image, application 1
